# Sustainable Delivery of Speech-language Therapy Services in Small Island Developing States Using Information and Communication Technology – A Study of the Maldives

**DOI:** 10.5195/ijt.2020.6306

**Published:** 2020-06-30

**Authors:** Mariyam Z. Zahir, Anna Miles, Linda Hand, Elizabeth C. Ward

**Affiliations:** 1 The University of Auckland, Auckland, New Zealand; 2 The University of Queensland School of Health and Rehabilitation Sciences, Brisbane, Queensland, Australia; 3 Centre for Functioning and Health Research, Metro South Hospital and Health Service, Brisbane, Queensland, Australia

**Keywords:** Children, Information and communication technology, Majority world countries, Service delivery, Speech-language therapy, Underserved communities

## Abstract

Small Island Developing States (SIDS), a subgroup of Majority world countries, face complex challenges providing equitable access to speech-language therapy (SLT) services. Increasing use of information and communication technology (ICT) to enhance SLT services is seen in the Minority world. This study explored the potential of using ICT to provide sustainable SLT services in one SIDS, the Maldives. A mixed method approach was used integrating data from (a) 21 online documents, (b) interview with an ICT official, and (c) surveys of 13 island councillors and 73 parents of children with communication difficulties. Almost 100% of the population had access to mobile phones and mobile broadband internet. Most parents were active and frequent ICT users. The government provided financial aid for people with disabilities which could be utilised to access ICT for services. Asynchronous service delivery using accessible ICT and parents as agents of service delivery can potentially enhance SLT services.

Equitable service provision for people with disabilities has been on the forefront of disability related dialogues since the publication of the ‘World Report on Disability’ in 2011 (World Health Organization & The World Bank, 2011). Yet service delivery for people with disabilities continues to be inadequate and lack consistency in many underserved communities (Cheng, 2013). While communication is recognised as a human right (United Nations, 1948), people with communication difficulties are particularly disadvantaged due to the way disability is frequently conceptualised and measured (Wylie, McAllister, Davidson, & Marshall, 2013). As a result, those with communication difficulties are not adequately represented among people with disabilities, leading to insufficient rehabilitation and support services for this population (Wylie et al., 2013). Disparities in access to services for children and adults with communication difficulties are more prominent between Majority world (previously referred to as developing countries) and Minority world countries (previously referred to as developed countries) (Cheng, 2016).

Traditional speech-language therapy (SLT) services for people with communication difficulties are individually focused or disorder-based (Wylie et al., 2013). These approaches are based on the assumption that the people who require services will seek and receive services equitably (McAllister, Wylie, Davidson, & Marshall, 2013). As the main priority of individually focused services is to manage individuals who reach their caseloads, they fail to meet the needs of the community as a whole (McAllister et al., 2013). Thus, these forms of traditional SLT service delivery largely fail to meet the demands of underserved communities (McAllister et al., 2013).

Wylie and colleagues (2013) consider a community to be underserved if SLT services do not exist, are inaccessible, or are not fairly distributed according to the needs of the population. These authors highlighted that the most frequently used approach in Minority world countries might not be the best or the most appropriate approach for all communities. Hence, they cautioned against the direct importation of service delivery approaches from Minority world countries to Majority world countries. The vastly different context, culture, language, and disability perceptions make the service delivery approaches of the Minority world inapplicable to the Majority world (Buell, 2013). Wylie and colleagues (2013) encouraged SLTs to think creatively and develop novel service delivery approaches that would address the needs of underserved communities and achieve equity in service provision.

A growing number of SLTs in Minority world countries have been adopting information and communication technology (ICT) to expand their services and improve service provision to their rural and remote communities (Molini-Avejonas, Rondon-Melo, Amato, & Samelli, 2015). Telepractice delivery is such an approach where traditional SLT services are provided using ICT in these settings (American Speech–Language–Hearing Association, 2016; Keck & Doarn, 2014; Speech Pathology Australia, 2014). In recent years, Majority world countries have also undergone considerable technological developments (International Telecommunication Union, 2017a). McLeod (2018) indicated that technology could be one of the solutions to support the rights of people with communication difficulties.

Novel ICT-based models have been explored in some underserved settings. Many rural and remote communities in China have compromised access to health care, and Cheng (2013) proposed three innovative models that could be adapted to address these challenges, one of which utilised ICT. Their project used mobile devices such as smart phones and mobile broadband-enabled computers to connect rural and urban health clinics in China. The project provided health care workers with an application that allowed access to patients' confidential electronic health records, educational and treatment material designed to address the needs of rural doctors and allowed remote consultations between rural and urban doctors. Since 2011, the project has expanded to 21 clinics and 150 doctors benefiting 160,000 patients. Cheng (2013) stated that this model can be modified and adapted to include SLT services in underserved and unserved communities.

Many innovative service delivery approaches that integrate ICT in underserved communities fail to be sustainable beyond their pilot phase (Fanta, Pretorius, & Erasmus, 2015). For this reason, ICT-based services are proposed to have a better chance of being successful if they are integrated into the existing conditions and technologies (Fanta et al., 2015). According to Fanta and colleagues (2015), sustainability of ICT-based services is determined by the environmental, social, and economic conditions of the underserved context (Fanta et al., 2015).

The environmental, social, and economic conditions in Majority world vary significantly to those of the Minority world (Fanta et al., 2015). Small Island Developing States (SIDS), a subgroup of 52 Majority world countries, face particularly complex environmental, social, and economic challenges related to their small size, remoteness, and vulnerability to natural disasters and climate change (Fontaubert, 1995). The challenges SIDS face have direct impacts on the integration and development of ICT in their communities. For instance, their small size affects market behaviour, remoteness influences cost of connectivity, and vulnerability to natural disasters requires infrastructure to be more resilient to destruction (Internet Society, 2017). As SIDS have made great progress in connectivity despite these challenges (Internet Society, 2017), exploring the conditions around ICT in these countries could help develop sustainable service delivery approaches in communities with similar complex characteristics.

The Maldives is a SIDS in the Indian Ocean made up of 1192 islands (Asian Development Bank, 2015). This archipelagic country covers an ocean area of more than 800 kilometres(km) long and 130km wide (Asian Development Bank, 2015). Ninety-nine percent of this area is covered by the ocean with just 1% of land (Ministry of Environment and Energy - Maldives, 2017). There are 26 naturally formed atolls (rings of islands) in the Maldives which the Maldivian government has divided into 20 administrative atoll areas (Asian Development Bank, 2015). A third of the 402,071 residents of the Maldives live in the capital island, Malé while the rest are scattered across 187 islands in 20 atolls (National Bureau of Statistics - Maldives, 2015). Wide disparities in development exist between Malé and the rest of the country. Most islands have established basic facilities like electricity, clean water, and health centres, but inequities exist when a wider variety of services and their quality is considered (Asian Development Bank, 2015). A total of 11 SLTs registered to practice in the Maldives from the beginning of allied health registrations in 2015 till the end of 2018 (Maldives Allied Health Council, 2017, 2018), and there was no indication of any service delivery outside of Malé.

These contextual factors need to be considered if the aim is to develop sustainable innovative service delivery approaches. This study was conducted as part of a bigger project that explored different contextual factors that could influence sustainable SLT service delivery in the Maldives. If we are to consider ICT as part of the potential solution to provide sustainable services, the specific contexts around ICT should also be examined. Hence, the current study explored the environmental, social, and economic conditions around ICT in the Maldives. The conditions that were explored are presented in Figure 1. In order to target the more underserved community, we decided to direct our attention to the two thirds of the population living in atolls outside the capital island, Malé. The focus of this study was services for children with communication difficulties as untreated communication difficulties in children could negatively impact their educational and socioemotional outcomes and result in pervasive difficulties through adulthood (Doell & Clendon, 2018; Elbro, Dalby, & Maarbjerg, 2011).

**Figure 1.**
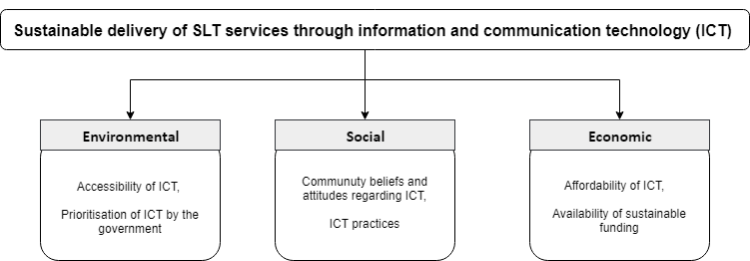
Environmental, social, and economic conditions that could guide the development of sustainable SLT service delivery approaches using ICT in SIDS.

## METHOD

This study received ethical approval from the University of Auckland (University of Auckland Human Participants Ethics Committee reference no. 020193) and approval to collect data from schools through the Ministry of Education of the Maldives.

For complex issues, using a single data collection method has potential to restrict the amount and type of information gathered, and lead to an incomplete understanding of the issue under investigation. Hence, we adopted a parallel convergent mixed-methods approach, incorporating interview, survey and document analysis methodologies, to provide a more comprehensive understanding of the research topic than would be collected from each of the components alone (Tashakkori & Teddlie, 2008).

**Figure 2.**
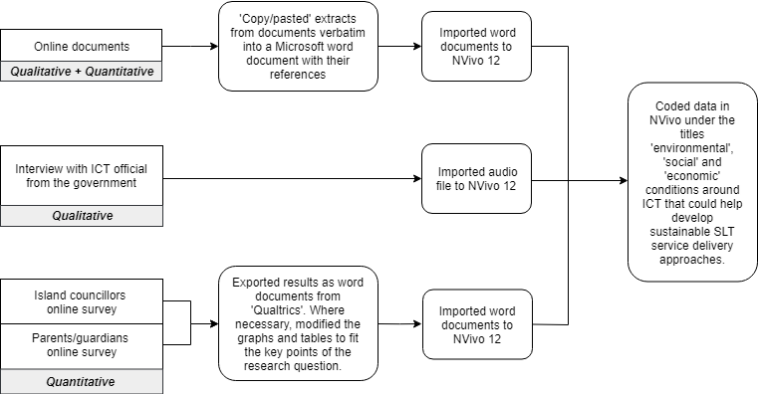
Mixed method data collection and analysis process.

Figure 2 displays the sequence of data gathering and analysis followed. The main sources of data included: (a) a review of publicly available online documents, (b) an interview with a government ICT official, and (c) online surveys conducted with island councillors and parents of children with communication difficulties in the Maldives. Data from all three sources were analysed and interpreted together to develop a better understanding of the conditions around ICT in the Maldives, which could help create sustainable ICT-based SLT service delivery approaches.

### DOCUMENT ANALYSIS

Document analysis was used to gather information about the context and to supplement and corroborate data collected from other sources (Bowen, 2009). We collected publicly available online documents by using internet searches. The search terms that were used included ‘Technology Maldives', ‘ICT Maldives', ‘Internet Maldives', ‘ICT policy Maldives', ‘ICT prices Maldives' and ‘Internet prices Maldives'. We also used reference lists of the documents discovered to find other relevant sources. Documents gathered from official websites that relayed authenticity were favoured over documents from unofficial sources such as newspaper articles and blog posts. Documents from both English and Dhivehi, the native language of the Maldives, were accepted. The steps outlined by Bowen (2009) were followed to collect and analyse the documents: (1) skimming or superficial examination, where the predefined codes ‘environmental', ‘social', and ‘economic' conditions were used locate any meaningful and relevant excerpts from the documents (2) reading and thoroughly examining the document, (3) interpretation, where we looked at excerpts to establish meaning and its contribution to the research question.

A total of 21 documents were retrieved for this study. Documents were collected from a diverse range of sources, though most were from the Maldivian government (Communications Authority of Maldives), international institutions (e.g., International Telecommunication Union) and official company websites. Appendix 1 includes all the documents that were gathered for this study. The documents provided both quantitative data (e.g., Telecom Statistics, ICT prices) and qualitative data (e.g., policies and regulations).

### INTERVIEW WITH GOVERNMENT ICT OFFICIAL

The purpose of the interview with government ICT official was to confirm the information collected through the document analysis and fill in gaps in information that were not discovered through documents. A government ICT professional was chosen for this purpose to gather perspectives from an official source who had access to information related to the Maldives. The interview included questions regarding the ICT services available in the country, disparities in access to ICT within the country, common uses of ICT by the community and challenges or needed improvements of ICT in atolls (Appendix 2).

In order to invite a government official, an email was sent to the main government agency that develops and promotes ICT in the Maldives. One government official who works in a position that would allow him to represent the agency and is well-informed about its operation was invited to participate in an interview for this study. The interview was conducted in Dhivehi.

Prior to analysis, the primary researcher translated the interview quotes from Dhivehi to English. The researcher reviewed the translated quotes with a second native speaker of Dhivehi who was fluent in both English and Dhivehi and was not involved in the research. To fully capture the expressed meaning, the intended meaning and its context in source language were discussed and where necessary, possible alternative words and phrases were deliberated.

### ONLINE SURVEYS

The third data source was the online surveys which were conducted to provide context to the information collected from other sources and gather insight on the actual use of ICT and the attitudes towards ICT by the population of interest. Online surveys allowed us to efficiently gather information and give voice to people in geographically dispersed locations across the Maldives (Northcott, Simpson, Moss, Ahmed, & Hilari, 2017; Ponto, 2015). The research team developed purpose-built online surveys and distributed them to (a) parents of children with communication difficulties and (b) island councillors of the Maldives.

#### SURVEY DESIGN

Both surveys were created and distributed through Qualtrics® (Qualtrics Survey Software LLC) and included multiple choice and short answer questions. Some sections of these surveys were out of the scope of this study since they were conducted as part of a larger project that explored other aspects of equitable and sustainable SLT service delivery. Appendix 3 provides the questions of the surveys that relate to this study.

Participants were provided with an English and Dhivehi version of the surveys in order to support any differences in language preference. To ensure that the meanings expressed in the English version were portrayed in the Dhivehi version, two to three native speakers who were fluent in English and Dhivehi and were not involved in the research fully checked the two versions before distribution. Following these reviews, the primary researcher amended the surveys as necessary. The survey completion times were between 10 to 15 minutes.

In order to target parents of children with communication difficulties, eligibility questions were included at the beginning of the parents' survey. This question included a list of communication skills (speech sounds, talking, hearing, reading, writing etc.) and asked parents whether they had any concerns about their child/children related to these skills. Access to the rest of the survey were only given if they answered yes. In this survey, we requested information regarding access to ICT, the quality of internet they receive, frequency of ICT use, purposes ICT was used for and their confidence to use ICT and fix any problems related to them.

In the island councillors' survey, we requested information regarding the percentage of people who had access to ICT in their islands, availability of public places to use ICT, availability of devices that can be used for public services and quality of internet their island receives.

#### SURVEY DISTRIBUTION

Contact details of 200 government schools located in all 187 inhabited islands, excluding Malé, were provided by the Ministry of Education of the Maldives for this study. The government schools of the Maldives are structured to begin at the foundation stage (aged 5 years) and continues till grade 10 or 12. Parents' survey was distributed electronically to all schools and the principals were requested to circulate them. The software used to distribute these surveys allows access to them through smartphones or computers. We requested principals to allow parents to use school computers if needed. Parents from across all regions of the Maldives outside Malé responded to the surveys. Characteristics of parents who completed the survey is presented in Table 1. Ninety percent of parents who participated were mothers or fathers and 10% were other family members.

**Table 1.** Characteristics of Parents and Island Councillors who Completed the Surveys

	Parents (n=73)	Island councillors (n=25)
	Number (Percentage)	Number (Percentage)
Gender		
Male	15 (21%)	17 (68%)
Female	58 (79%)	8 (32%)
Age		
under 20	0 (0%)	0 (0%)
20-30	29 (40%)	9 (36%)
31-40	39 (53%)	6 (24%)
over 40	5 (7%)	10 (40%)
Ethnicity		
Maldivian	66 (97%)	25 (100%)
Indian	1 (1%)	0 (0%)
Other	1 (1%)	0 (0%)

The local government authority provided us with a list of emails for all 200 island and city councils of the Maldives. The survey was distributed electronically to all island and city councils outside Malé. One island councillor from each council was invited to complete the online survey. Table 1 below shows the characteristics of island councillors who completed the surveys. Thirteen percent of island councillors of the Maldives responded to the survey which included councillors from all regions and from all five population size classes (less than 500, 500-999, 1000-5000 and more than 5000 people).

### DATA ANALYSIS

Data from all sources were analysed and interpreted using NVivo 12 which supports analysis of mixed method studies (QSR International, Melbourne, Australia) (Figure 2). As NVivo 12 software allows researchers to import and synthesise different types of quantitative and qualitative data (QSR International, 2017), we were able to manage data sets from all three sources in one platform. Content analysis was used to code data which requires gathered information to be organised into categories related to the research questions (Bowen, 2009). Data from all sources were categorised into ‘environmental', ‘social', and ‘economic' conditions around ICT in the Maldives. The coded sections were further categorised into the eight areas explored within each of the main categories. The first author is fluent in both languages and hence initially analysed all data which was followed by the second and third author checking the analysed data. The research team corrected errors to ensure categories and subcategories corresponded with the raw data and consensus was gained where there was disagreement.

## RESULTS

The synthesised data from the documents, interview and surveys are presented below under the three main categories: ‘environmental conditions', ‘social conditions', and ‘economic conditions' and their subcategories.

### ENVIRONMENTAL CONDITIONS

#### ACCESSIBILITY OF ICT

The Maldives has two national fibre-optic submarine cable networks of over 1200kms each, running across the length of the country (International Telecommunication Union, 2018). These networks support fixed broadband and mobile broadband services (International Telecommunication Union, 2018). Fixed broadband refers to high speed bandwidth transmission to fixed locations like homes and businesses by using cables, fibre optics, wireless and other technologies, and mobile broadband refers to wireless internet access through mobile phone towers to mobile devices such as portable modems, tablets and mobile phones (International Telecommunication Union, 2006). The two national cables are laid by the two telecommunications providers of the Maldives, DHIRAAGU and Ooredoo (DHIRAAGU, 2018; Ooredoo Maldives, 2017). According to the government ICT official, the Maldivian government equally distributed provision of services to allow each service provider to cover 50% of the country.

The telecommunications providers in the Maldives offer fixed fibre broadband with speeds up to 1Gbps (DHIRAAGU, 2019). DHIRAAGU provides high-speed fibre for the home (FTTH) services to 55 islands that represent 74% of national households, while Ooredoo provides fixed broadband to 20 islands and FTTH over 10 islands (DHIRAAGU, 2018; Ooredoo Maldives, 2018; Telegeography, 2018). Though high speed fixed broadband internet is accessible to the majority of the country, according to the government ICT official, the quality and speed of the internet they receive are determined by the budget the island council receives by the government:

“Currently councils are likely to get a better budget if the islands are bigger. So, these islands can access better internet connections.” (ICT official)

In addition to this, some islands receive intermittent fixed broadband connections due to electrical voltage fluctuations (Ministry of Education - Maldives, 2019). These fluctuations cause damage to wired internet requiring replacements of ethernet switches and cause wireless internet disconnections as Wi-Fi access points are left unpowered (Ministry of Education - Maldives, 2019). However, according to the ICT government official internet is quite stable across the country because of fibre cables, as they no longer lose connections due to weather conditions. The official stated that previously when microwave connections were used people experienced disconnections as a result of bad weather. It was also noted that the only reason to lose connectivity now is due to broken network cables caused by roadworks.

Compared to fixed broadband internet, mobile broadband internet appears to be more accessible across the Maldives (Table 2). Since 2018, DHIRAAGU claims to provide high-speed 4G mobile broadband internet services to 100% of the population across all inhabited islands (DHIRAAGU, 2018). This was confirmed by the government official who noted that all islands where people reside receive at least 3G mobile broadband connections.

**Table 2.** Access to ICT in the Maldives

	Malé	Atolls	Maldives
ICT availability in households^1^ (%)			
Electricity	100	100	100
Internet	73.4	63.4	67.2
Computers	86.9	59.8	70.1
Mobile phones	98.6	98.8	98.7
Fixed phone lines^2^			
Total number of fixed phone lines (includes payphones)			18,457
Teledensity (%)			4.9
Mobile subscriptions^2^			
Mobile subscribers			842,683
Post-paid			148,965
Pre-paid			693,718
Teledensity (%)			227.9
Internet subscriptions^2^			
Fixed broadband subscribers			48,455
Mobile broadband subscribers			282,718
Internet Speed^3^			
Fixed broadband internet speed			16.27 Mbps
Mobile internet speed			21.68 Mbps

1 (Ministry of Health - Maldives and ICF 2018

2 Communications Authority of Maldives 2019

3 Speedtest 2019

*Note.* The figures presented in any single row of the table might not be directly comparable to others as a result of using different sources that were updated on different dates, ranging from 2018 to July 2019.

**Figure 3.**
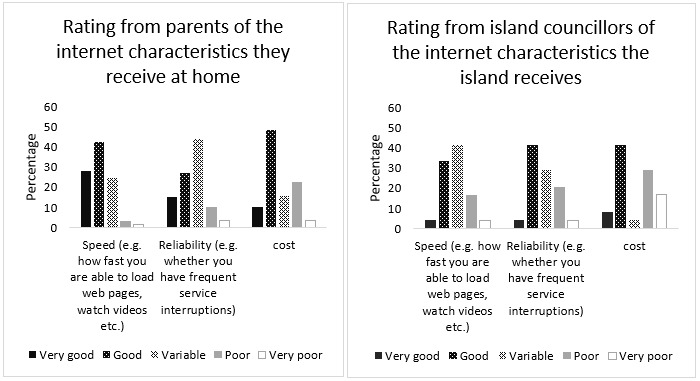
(a). Rating of internet characteristics accessed at home as indicated by parents. (b). Rating of internet characteristics accessed in islands as indicated by island councillor

Access to ICT in the Maldives as revealed by documents is provided in Table 2. From the surveyed parents, 91% had access to internet while surveyed island councillors estimated an average of 80% of the island populations to have access to internet. Ninety-six percent of women and 97% of men between the ages of 15-49 years own a mobile phone in the Maldives (Ministry of Health - Maldives & ICF, 2018). Ninety-nine percent of the surveyed parents said they had access to mobile smart phones while surveyed island councillors estimated an average of 89% of the island population to have access to mobile smart phones. The satisfaction ratings of the quality of internet received by these parents and island councillors can be seen in Figure 3.

Fifty-nine percent (n=10) of parents who had difficulties fixing ICT problems on their own noted having access to someone at home who can fix problems they experience with internet and other ICT. Seventy-one percent (n=17) of island councillors stated having services or a person present in their island to fix public problems with internet and other ICT, at least occasionally. When enquired about public access to internet, 75% (n=18) of island councillors indicated that they have no public places like cyber cafes where people can access internet and other ICT.

In addition to ICT integration in the general public, in 2001 the Asian Development Bank (ADB) established the Government Network of Maldives, a fibre optic network that interlinks government agencies in Malé and other islands (Asian Development Bank, 2012). According to the government ICT official, a national computer network was established around 6 years ago which connected all ministries, government hospitals and schools in Malé. The official also stated that all island councils are connected using lease lines which allows them to access government's web applications or web portals. As the next phase, it was indicated that plans are in place to connect schools and health centres of islands through island councils.

#### PRIORITISATION OF ICT BY THE GOVERNMENT

ICT related national policies, laws and regulations have been administered in the Maldives since 2001. The second telecommunication policy enacted in 2006 included policies such as providing high speed internet services throughout the country and establishing reasonable communication means for people with disabilities (Ministry of Transport and Communication - Maldives, 2006). The National Broadband Policy enacted in 2014 included policies such as maintaining the quality of broadband services and increasing the number of services provided through internet (International Telecommunication Union, 2018). Other notable actions include the enactment of the Maldives Telecommunication Law in 2015 and the establishment of National Centre for Information Technology (NCIT) as the main government agency for the development, promotion and propagation of Information Technology in the Maldives (National Centre for Information Technology, 2012). Details of notable ICT policies, laws and regulations are provided in Appendix 4.

According to the ICT government official, the Maldivian government has not yet provided many ICT services for citizens. The official stated that the main role of National Centre for Information Technology is to support the government's ICT needs as necessary. However, it was also mentioned that there were some initiatives such as the development of housing and expatriate job recruitment portals that citizens can use.

The government official stated that the government fixes internet connection interruptions caused by broken fibre cables in islands within a week or two while noting that it takes time to travel and fix these issues in islands. The official indicated that they have service contracts with service providers to provide aid in such situations.

The government official expressed concern regarding the lack of people with technical experience in the government which hinders ICT development in the country:

“Government doesn't employ a lot of technical people. I don't see them trying to build capacity within the government. Private sectors have more experienced people. People might not want to join the government because of the civil service pay scale.” (ICT official)

However, the official was also hopeful about the government's involvement in future ICT developments of the Maldives:

“I'm hoping these things will improve in the next 5 years. Since we are under a new ministry now (Ministry of Communication, Science and Technology), we can represent ICT in the cabinet level as well. We were previously under the finance ministry. They have no relation to ICT. So even if we bring up an issue, it was not much of a concern to them. But now I believe things will change. I see positive changes in the next 5 years.” (ICT official)

### SOCIAL CONDITIONS

#### ICT PRACTICES

The number and proportion of active internet users, mobile internet users and social media users are provided in Table 3. The top 5 websites accessed in the Maldives were all local news websites (Alexa, 2019).

**Table 3.** ICT use in the Maldives

Internet and social media users	Number	Percentage of the population (%)
Active Internet users^3^	370,000	83
Active mobile internet users^4^	350,000	78
Active social media users	370,000	83
Active social media users accessing via mobile devices	350,000	78

3 Hootsuit & We Are Social, 2019

4 Internet World Statistics, 2019

**Figure 4.**
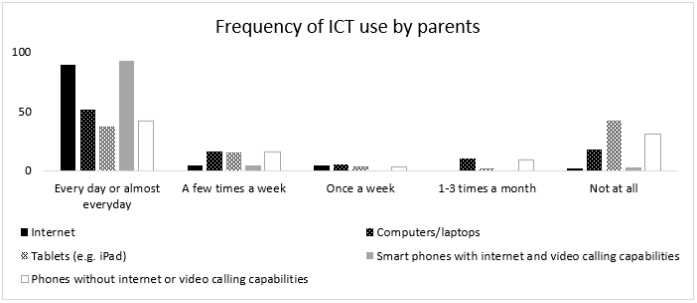
Frequency of ICT use by parents in their daily lives.

Figure 4 presents the frequency of ICT use by surveyed parents in their daily lives. Table 4 provides the common uses of ICT by these parents.

**Table 4.** Internet and ICT uses by Parents

Uses	Percentage of respondents (%)	Proportion of Maldivian sources (%)	Proportion of sources outside the Maldives (%)
To collect information about different topics of interest	84	58	42
For work projects and purposes	63	54	46
To access information and advice related to their children (e.g. behaviour, medical, health etc.)	71	57	43
To access information and advice about speech and language difficulties in children	58	58	42
For entertainment (e.g., playing games, watching movies, etc.)	73	43	57
To communicate with others, using:	78	71	29
Emails	34		
Instant messaging (e.g. Viber)	74		
Video calls (e.g., Skype)	19		
Other means (included phone calls, Facebook and Instagram)	15		
To connect with different professionals with concerns regarding their child/children.	49	74	26
Professionals included:			
Doctors/nurses	27		
Special education teachers	22		
Therapists	7		
Other (included family, friends, psychologists, and religious scholars)	11		

#### COMMUNITY BELIEFS AND ATTITUDES REGARDING ICT

**Figure 5.**
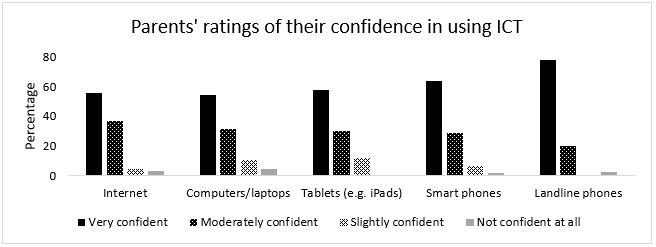
Parents' rating of their confidence in using ICT.

The confidence ratings of parents to use internet and other devices are provided in Figure 5.

Fifty-one percent of surveyed parent noted it being sometimes easy to fix ICT problems on their own, while 5% of parents noted it being very easy. Only 28% of parents noted it being difficult or very difficult.

Regarding attitudes towards new ICT applications, the government ICT official expressed the importance of educating the public if they are to make good use of them:

“If we create an app and ask them (the public) to use it, to say, apply for their ID card and passport, they wouldn't know how to use it. We have to create awareness among people in islands. Not only in islands, even in Malé too. If I take MIRA (Maldives Inland Revenue Authority) for an example, ‘MIRA connect' can be used to pay tax, submit BPT reports and a lot of other things. But so many people are still physically going there and queuing up to do these tasks instead.” (ICT official)

### ECONOMIC CONDITIONS

#### AFFORDABILITY OF ICT

The average per capita monthly income in the Maldives is MVR4944 (USD321) (National Bureau of Statistics - Maldives, 2018). Per capita income is calculated by summing up the total income of a household and dividing it by the population of that household. Per capita income in Malé (MVR6,984 (USD453)) is more than twice the income in atolls (MVR3421(USD222)) (National Bureau of Statistics - Maldives, 2018). Table 5 below shows the ICT prices in the Maldives as a proportion of these incomes.

**Table 5.** ICT Prices and Prices as a Proportion of Monthly Incomes

ICT packages	MVR (USD)	Proportion of average income in Malé (%)	Proportion of average income in atolls (%)
Mobile-cellular prices (voice and SMS)	93.75 (6.08)	1	3
Mobile-broadband prices			
Prepaid handset-based plans (500MB)	105.32 (6.83)	2	3
Post-paid computer-based plans (1GB)	211.72 (13.73)	3	6
Fixed-broadband prices (unlimited - speed 4Mbit/s)	308.4 (20)	4	9

(International Telecommunication Union, 2017b)

The ICT government official expressed high cost of fixed broadband prices as a challenge in the Maldives:

*“I think internet is the biggest challenge at the moment. Cost for example. The cost is quite high in the Maldives.”* (ICT official)

Telecommunication providers in the Maldives are now offering prepaid deals that include free use of social media apps and the ability to customise voice and data add-ons (DHIRAAGU, 2018). The satisfaction ratings of internet cost by parents and island councillors are provided in Figure 3.

#### AVAILABILITY OF SUSTAINABLE FUNDING

Since 2018, the Ministry of Gender, Family and Social Services in the Maldives has been running a program that provides free therapeutic services for people with disabilities (Ministry of Gender Family and Social Services - Maldives, 2018). Under the ‘Disability act of the Maldives' enacted in 2010, people with disabilities are also eligible to receive a disability allowance of MVR2000 (~USD130) per month (National Social Protection Agency - Maldives, n.d.). However, approximately half the people with disabilities are receiving this allowance at the moment (Ministry of Health - Maldives & ICF, 2018).

## DISCUSSION

We explored the conditions around ICT in the Maldives that could help create sustainable ICT-based SLT service delivery approaches. The study revealed that there was good access to ICT across the Maldives, with almost 100% of the population having access to mobile phones and mobile broadband internet, indicating favourable environmental conditions to use ICT for sustainable SLT service delivery. There was a large number of active internet users across the Maldives who frequently used ICT for a variety of purposes, displaying promising social conditions for sustainable delivery of SLT services through ICT. While the cost of fixed broadband was higher compared to mobile broadband, the government provides financial aid and free therapy for people with disabilities, suggesting some economic conditions that could facilitate the development of sustainable SLT services delivery using ICT.

Some of the favourable environmental conditions we discovered are however subject to caveats. Even though all Maldivian households had access to electricity and widespread access to internet, mobile phones and computers, strong fixed broadband services were not accessible in most islands. The fibre network cables that extend across the country would allow access to high speed fibre internet, but currently the quality of the fixed broadband they receive is determined by the budget the island receives from the government. While prioritisation of ICT by the government could be the reason for the high general access of ICT in the Maldives, some aspects of the policies have not yet been put into practice (e.g., providing high speed internet services throughout the country and maintaining the quality of broadband services).

In contrast, strong mobile broadband services are accessible throughout the country. Mobile broadband connections can be purchased by individuals without relying on government funds for the islands. In most SIDS, mobile broadband has largely stepped in to compensate for limited fixed broadband access (Internet Society, 2017). The rapid growth of mobile broadband and mobile communication in SIDS could make services more cost effective for citizens and efficient for the government by enabling online and mobile-based public service delivery (Internet Society, 2017). The ICT model proposed by Cheng (2013) used 3G mobile broadband with mobile phones and mobile broadband-enabled computers in underserved communities to provide equitable services. As almost all Maldivian households possess mobile phones, such service delivery approaches that use mobile phones with mobile broadband connections are likely to be plausible in the Maldives and other SIDS with similar ICT conditions. Since 60% of households in Maldives also owned computers, mobile broadband-enabled computers could be considered as a means for service delivery as well.

Even though high-speed mobile broadband is accessible, reliability of internet is of concern as more than 50% of surveyed parents and island councillors rated reliability between ‘variable' to ‘very poor'. This makes synchronous (real-time) service delivery approaches less likely to be successful. To compensate for such internet instabilities, Minority world countries have used a hybrid of synchronous and asynchronous (store and forward or delayed delivery) approaches while using telepractice approaches to provide traditional SLT services (Keck & Doarn, 2014). SIDS such as the Maldives might be required to develop novel approaches of service deliveries that rely solely on asynchronous methods.

The favourable social conditions centred around the high proportion of active internet users and high frequency of ICT use in the Maldives. A majority of the parents who responded to the survey used internet and mobile smartphones every day or almost every day and rated themselves as being ‘very confident' to ‘moderately confident' in using them. These parents used internet and ICT for a variety of purposes including to access information and advice related to their children. The results provide evidence of ICT familiarity, and flexibility and confidence to use ICT, especially among parents of children with communication difficulties in the Maldives. Hence, this population would be worth looking into as recipients of intervention or agents of service delivery if an ICT-based SLT service delivery approach is considered in the Maldives. Wylie and colleagues (2013) presented ‘recipients of intervention' and ‘agent of delivery of intervention' as aspects of service delivery that would influence the choice of approaches to use in different contexts. Immediate circle including family was suggested as ‘recipients of intervention' and family members such as parents and siblings guided by SLTs was suggested as ‘agents of delivery'.

While parents are confident to use ICT devices, the ICT official indicated that the public does not always adopt new ICT applications. Education and training might be required if new applications are used for SLT service delivery. As services are more likely to be sustainable in underserved communities if integrated into current practices (Fanta et al., 2015), perhaps a more successful approach would be to employ ICT practices that the community is already familiar with using. Seventy-four percent of the parents were already using instant messaging to communicate, and some were connecting with professionals such as doctors and special education teachers with concerns regarding their children. Thus, instead of introducing new applications, instant messaging can be expected to have a better chance of success as a new medium of SLT service delivery.

The economic conditions we explored unearthed some barriers and facilitators to providing sustainable SLT service delivery using ICT. Internet costs were considered a challenge by the ICT official. The high fixed broadband prices result in islands only being able to afford low bandwidths leading to inadequate fixed broadband access. Fixed broadband prices were three times higher than mobile broadband and cellular prices, as a proportion of their income in atolls. However, 68% of the surveyed parents rated their satisfaction of internet prices as ‘very good' or ‘good'. Hence, we can assume that for a majority of the population, cost associated with accessing internet is not an issue. Furthermore, the Maldivian government provides a monthly disability allowance of MVR2000 for people with disabilities. If children with communication difficulties are provided with this allowance, mobile broadband plans can be purchased by using 5% of this allowance. The Maldivian government has also launched a program which provides free therapeutic services for people with disabilities. If SLT services are not directly accessible in atolls, there is scope for the Maldivian government to consider providing compensations for the prices associated with accessing alternative service delivery approaches.

The conditions around ICT in the Maldives might not be suitable to provide services through telepractice approaches similar to those provided in Minority world countries. However, the access, use, attitudes, and costs of ICT identified in this study gives hope to novel ICT-based service delivery approaches that can sustainably provide equitable SLT services in the Maldives. Such approaches developed in SIDS might be applicable in other Majority world countries as well. As many Minority world countries are failing to meet the needs of their rural and remote regions, these countries might also benefit from the new approaches developed in Majority world contexts (Wylie et al., 2013).

## LIMITATIONS

As parents' and island councillors' surveys were conducted online, a bias towards participants who had superior ICT access needs to be considered as an influencer in the data collected. Additionally, since the proportion of island councillors who responded to the survey were fairly low, it might not represent views of the whole population. However, the island councillor participants represented all regions and population classes of the Maldives. Even though these were considered limitations, using a mixed method approach allowed us to verify and cross-check data from multiple sources and gather comprehensive information related to the research questions. This provided more credibility to the overall results that were presented in our study.

## CONCLUSION

The aim of this research was to explore the conditions around ICT in the Maldives in order to examine ICT as part of the solution to provide sustainable SLT service delivery approaches. As SIDS face complex environmental, social, and economic challenges and these factors also determine sustainability of ICT-based service delivery approaches, we explored these conditions in the Maldives. We focused on the more underserved community who live outside the capital island Malé and targeted services for children with communication difficulties. The key findings of this study include (a) The Maldivian population has good access to ICT. Almost 100% of the population have access to mobile phones and mobile broadband across the country, (b) Parents of children with communication difficulties were found to be active internet users who frequently use ICT confidently for a variety of purposes, and (c) The cost of fixed broadband is higher compared to mobile broadband. However, the government provided financial aid for people with disabilities which could be utilised to cover expenses related to ICT-based services, in cases where services are not locally available.

The results of the study direct sustainable SLT service delivery in the Maldives to utilise parents as agents of service delivery to deliver asynchronous SLT services by using mobile broadband internet, mobile devices, and instant messaging. Further research needs to be conducted to identify other aspects such as the focus of intervention (impairment, activity/participation, etc.) and level of intervention (prevention, early intervention, rehabilitation, etc.) which would help develop sustainable service delivery approaches that meet the demands of the community. As this study explored the environmental, social, and economic conditions, other SIDS may be able to design sustainable ICT-based SLT service delivery approaches in their communities by following these steps. Through the development of innovative service delivery approaches that are tailored to these underserved communities, people with communication difficulties in these communities can be provided with the support to exercise their right to communicate.

## APPENDIX 1 DOCUMENTS RETRIEVED IN THE DOCUMENT ANALYSIS

**Table:**

Source document			Document type
**Environmental aspect**			
Accessibility of ICT	1.	Maldives: Information Technology Development Project (South Asia Project Brief) (Asian Development Bank, 2012)	Institutional report
	2.	Telecom Statistics - 2019 Monthly Figures (Communications Authority of Maldives, 2019)	Government statistical release
	3.	Dhivehi Raajjeyge Gulhun Annual Report 2018 (DHIRAAGU, 2018)	Telecommunications provider report
	4.	Measuring the Information Society Report Volume 2. ICT Country Profile (International Telecommunication Union, 2018)	UN agency report
	5.	Asia Internet Usage Stats Facebook and 2019 Population Statistics (Internet World Statistics, 2019)	World internet usage statistics website
	6.	Education Sector Analysis Maldives (Ministry of Education - Maldives, 2019)	Government report
	7.	Maldives Demographic and Health Survey 2016-17 (Ministry of Health - Maldives & ICF, 2018)	Government report
	8.	Ooredoo's Nationwide Submarine Cable Lands in Thinadhoo! | Ooredoo.(Ooredoo Maldives, 2017)	Telecommunications provider article
	9.	Speedtest Global Index – Monthly Comparisons of Internet Speeds from around the World (Speedtest, 2019)	Global internet speed testing website
	10.	Digital 2019 - Maldives (January 2019) (Hootsuit & We Are Social, 2019)	Social-media management platform report
Prioritisation of ICT by government	1.	Maldives Telecommunications Regulation 2003 (Communications Authority of Maldives, 2003)	Government regulations document
	2.	Information and Communication Technology in the Atolls: Maldives Case Study (International Telecommunication Union, 2004)	UN agency report
	3.	Measuring the Information Society Report Volume 2. ICT Country Profiles (International Telecommunication Union, 2018)	UN agency report
	4.	Maldives Telecommunications Law 2015 (Maldives Government Gazette, 2015)	Government law official document
	5.	Maldives Telecommunication Policy 2006-2010 (Ministry of Transport and Communication - Maldives, 2006)	Government policy official document
	6.	National Broadband Policy 2014-2018 (*National Broadband policy 2014-2018*, n.d.)	Government policy official document
	7.	National Centre for Information Technology - About Us (National Centre for Information Technology, 2012)	Government agency website
**Social aspect**			
ICT practices	1.	Digital 2019 - Maldives (January 2019) (Hootsuit & We Are Social, 2019)	Social-media management platform country report
	2.	Alexa - Top Sites in Maldives – Alexa (Alexa, 2019)	
**Economic aspect**			
Household income	1.	ICT PRICES (International Telecommunication Union, 2017b)	UN agency report
	2.	Household Income and Expenditure Survey (HIES) Analytical Report: Household Income 2016 (National Bureau of Statistics - Maldives, 2018)	Government report
Affordability of services	1.	Maldives Demographic and Health Survey 2016-17 (Ministry of Health - Maldives & ICF, 2018)	Government report
	2.	Inaugurating the provision of free therapeutic services for people with disabilities, as part of the current administration's first 30-day goals (Ministry of Gender Family and Social Services - Maldives, 2018)	Ministry website
	3.	Disability Allowance (National Social Protection Agency - Maldives, n.d.)	Government website
	4.	Dhivehi Raajjeyge Gulhun Annual Report 2018 (DHIRAAGU, 2018)	Telecommunications provider report

## APPENDIX 2 GOVERNMENT ICT OFFICIAL INTERVIEW GUIDE

1. Tell me about the ICT services available in atolls.
  - What about internet? (e.g., speed, reliability, band width and cost)?
  - What do you think are the common uses of ICT infrastructure in atolls?
  - In your opinion, what are the shortfalls, challenges or needed improvements of ICT services in atolls? (e.g., streaming of videos, looking at webpages)
2. Are there any differences or disparities across different atolls?
3. What developments would you like to see happen in atolls in the next five years?
  - What about developments related to educations/health?
  - What about charity initiatives and supports for health and education?
  - What about developments related to personal use, entertainment?
4. Do you have anything else to add regarding ICT services in the Maldives?

## APPENDIX 3 PARENT SURVEY: SECTIONS RELATED TO THIS STUDY

Eligibility questions to be answered prior to progressing into survey

1. International ethical approval requires all survey participants to be over 16 years old. Please confirm that you are over 16 before completing the survey.
  - I am 16 years or older
  - I am under 16 years of age.
  (If under 16 years is selected the following message will be displayed and the survey will end)
  Thank you for your interest in participating in the survey. However, we regret to inform that we are only able to collect data from people who are 16 years or over for this study.
2. Are you a parent of a child between 0-16 years of age?
  - Yes
  - No
  (If ‘No' is selected the following message will be displayed and the survey will end)
  Thank you for your willingness to participate in this survey. If you are not a parent of a child between 0-16 years of age but have an interest in participating in this study, please contact the researcher at mzah253@aucklanduni.ac.nz.
3. Do you have any concerns about any of your children with regards to talking, saying sounds, hearing, following spoken instructions, reading, writing, learning or social skills?
  - Yes
  - No
  (If ‘No' is selected the following message will be displayed and the survey will end)
  Thank you for your willingness to participate in this survey. If you do not have any concerns about your child's talking, saying sounds, hearing, following spoken instructions, reading, writing, learning or social skills, but have an interest in participating in this study, please contact the researcher at mzah253@aucklanduni.ac.nz.
4. Do you currently live in the Maldives?
  - Yes
  - No
  (If ‘No' is selected the following message will be displayed and the survey will end)

Thank you for your willingness to participate in this survey. If are a Maldivian who does not currently live in the Maldives but have an interest in participating in this study, please contact the researcher at mzah253@aucklanduni.ac.nz.

The following questions relate to various types of technology that you may use and are available for you.

1. Are the following available for you to use at home?
  **Table:**
  	Yes	No	Unsure
  Internet	○	○	○
  Computers/laptops	○	○	○
  Tablets (e.g. iPad)	○	○	○
  Smart phones with internet and video calling capabilities	○	○	○
  Phones without internet or video calling capabilities	○	○	○
2. How would you rate the following aspects of the internet you receive at home? *(displayed if ‘Yes' is selected for internet access at home in the previous question)*
  **Table:**
  	Very good	Good	Variable	Poor	Very poor
  Speed (e.g., how fast are you able to load webpages, watch videos etc.)	○	○	○	○	○
  Reliability (e.g., whether you have frequent service interruptions)	○	○	○	○	○
  Cost	○	○	○	○	○
3. How frequently do you use the following types of technology in your personal life?
  **Table:**
  	Every day or almost everyday	A few times a week	Once a week	1-3 times a month	Not at all
  Internet	○	○	○	○	○
  Computers/laptops	○	○	○	○	○
  Tablets (e.g., iPad)	○	○	○	○	○
  Smart phones with internet and video calling capabilities	○	○	○	○	○
  Phones without internet or video calling capabilities	○	○	○	○	○
4. How confident are you with using the following types of technology in your personal life? *(only statements where anything other than ‘Not at all' was selected in the previous question will be displayed)*
  **Table:**
  	Very Confident	Moderately confident	Slightly confident	Not confident at all
  Internet	○	○	○	○
  Computers/laptops	○	○	○	○
  Tablets (e.g., iPad)	○	○	○	○
  Smart phones with internet and video calling capabilities	○	○	○	○
  Phones without internet or video calling capabilities	○	○	○	○
5. How easy is it for you to fix problems related to internet or other Information and Communication Technologies (e.g., computers, laptops, tablets, smartphones etc.) on your own? *(displayed if anything other than ‘Not at all' was selected for any statement in question 13)*
  - Very easy
  - Sometimes easy
  - Neither easy nor difficult
  - Difficult
  - Very difficult
6. Do you have access to a person/people who can fix problems you experience at home with internet and other Information and Communication Technologies (e.g., computers, laptops, tablets, smartphones etc.)? *(displayed if ‘difficult' or ‘very difficult' is selected in the previous question)*
  - Yes
  - Occasionally
  - No
  - Unsure
7. For what purposes do you use the internet and devices (e.g., smartphones, tablets, computers etc.)? Please select all that apply. *(displayed if ‘Yes' is selected for any statement in question 11 AND anything other than ‘Not at all' is selected for devices or internet in question 13)*
  - To collect information about different topics of interest
    - From sources within the Maldives
    - From sources outside of the Maldives
  - For work projects and purposes
    - Using sources within the Maldives
    - Using sources outside of the Maldives
  - To access information and advice related to your children (e.g., behaviour, medical, health etc.)
    - From sources within the Maldives
    - From sources outside of the Maldives
  - To access information and advice about speech and language difficulties in children
    - From sources within the Maldives
    - From sources outside of the Maldives
  - For entertainment (e.g., playing games, watching movies etc.)
    - Using sources within the Maldives
    - Using sources outside of the Maldives
  To communicate with others using:
    - emails
      - To people within the Maldives
      - To people outside of the Maldives
    - instant messaging (e.g., Viber)
      - To people within the Maldives
      - To people outside of the Maldives
    - video calls (e.g., Skype)
      - To people within the Maldives
      - To people outside of the Maldives
    - other means of communication (please specify_____)
  To connect with the following professionals with concerns regarding my child/children:
    - Doctors/nurses
      - Within the Maldives
      - Outside of the Maldives
    - Special education teachers
      - Within the Maldives
      - Outside of the Maldives
    - Therapists (please specify ______)
      - Within the Maldives
      - Outside of the Maldives
    - Other (please specify ______)
  - Other purposes (please specify ____________)
8. For which of the following purposes would you like to use internet and devices (e.g., smartphones, tablets, computers etc.)? Please select all that apply. (*displayed if ‘No' is selected for access to all devices and internet in question 11 OR, if ‘Not at all' is selected for devices or internet in question 13)*
  - To collect information about different topics of interest
  - For work projects and purposes
  - To access information and advice related to children (e.g., behaviour, medical, health etc.)
  - To access information and advice about speech and language difficulties in children
  - For entertainment (e.g., playing games, watching movies etc.)
    To communicate with others using:
      - emails,
      - instant messaging (e.g., Viber)
      - video calls (e.g., Skype)
      - other means of communication (please specify_____)
    To connect with the following professionals with concerns regarding my children:
      - Doctors/nurses
      - Special education teachers
      - Therapists (please specify ______)
      - Other (please specify ______)
  - Other purposes (please specify ____________)

### ISLAND COUNCILLOR SURVEY: SECTIONS RELATED TO THIS STUDY

The following questions relate to various types of technology and related services that might be available in your island.

1. Approximately what percentage of people in your island have access to
  - Internet
  - Computers/laptops
  - Tablets (e.g., iPad)
  - Smart phones with video calling capabilities
  - Phones without video calling capabilities
2. Are there public places (e.g., cyber cafes) where people in your island can access internet and other Information and Communication Technologies (e.g., computers, laptops, tablets etc.)?
  - Yes
  - No
  - Unsure
3. How would you rate the following aspects of the internet you receive in your island? *(Displayed if anything above 0 is selected for internet access in question 6)*
  **Table:**
  	Very good	Good	Variable	Poor	Very poor
  Speed (e.g., how fast you are able to load webpages, watch videos etc.)	○	○	○	○	○
  Reliability (e.g., whether you have frequent service interruptions)	○	○	○	○	○
  Cost	○	○	○	○	○
4. Are there any IT services or a person in your island who can fix problems with internet and other Information and Communication Technologies (e.g., computers, laptops, tablets, smartphones etc.) for the people living in your island?
  - Yes
  - Occasionally
  - No
  - Unsure
5. Are there any devices such as computers, laptops, tablets, or smartphones in your island that can be made available for children with speech and language difficulties, their families or teachers, to access services?
  - Yes
  - No
  - Unsure
6. Where in your island can these devices be accessed to support children with speech and language difficulties, their families or teachers? Please select all that apply. *(Displayed if ‘yes' is selected for the previous question).*
  - Schools
  - Hospitals, health centres or clinics
  - Government offices
  - Private homes
  - Public areas (please provide a description_________)
  - Other (please specify________________)

## APPENDIX 4 NATIONAL ICT POLICIES AND REGULATIONS AND OTHER NOTABLE ACTIONS BY THE GOVERNMENT

**Table:**

National policies, laws. and regulations	Relevant details
2001 Telecommunication Policy	Policies included: To reduce charges of all telecommunication services Expand telecommunication services and to reduce disparities in service provision between Malé and the other islands. Open telecommunication sector and encourage competition Facilitate the use of info-communication technology in all areas of development” (International Telecommunication Union, 2004)
2003 ‘Maldives Telecommunications Regulation 2003' came into force and ‘Telecommunications Authority of Maldives (TAM)' was established as industry regulator (International Telecommunication Union, 2004)	Objectives of this regulation included: To promote the best interests of the citizens of the Maldives, and to create an environment conducive to investment, by: – ensuring that telecommunications services are accessible to all people in the Maldives and are supplied as efficiently and economically as practicable and at performance standards that reasonably meet the social and commercial needs of the Maldives. – encouraging, promoting, and facilitating the development and expansion of a telecommunications industry that is efficient, internationally competitive and responsive to the needs of the community. to promote a competitive environment for the provision of domestic and international telecommunication services”
	(Communications Authority of Maldives, 2003)
2003 National Centre for Information Technology (NCIT)	“Established by Government of Maldives as the main government agency for the development, promotion and propagation of Information Technology (IT) in the Maldives. NCIT is the government agency for the development, promotion and dissemination of Information Technology in the country” (National Centre for Information Technology, 2012)
2006 A second Telecommunications Policy was introduced	Policies included: Telecommunications charges shall be non-discriminatory, affordable and cost oriented Telecom infrastructure shall be expanded and developed to provide basic, enhanced and broadband services throughout the country – Provide basic telecommunications services to all – Introduce alternative technologies that provide high capacity to improve national connectivity – Provide high speed Internet services throughout the country – Establish reasonable communication means for people with special needs [PWDs] – The use and development of telecommunication technology shall be facilitated for the Maldives to fully embrace its benefits – Increase awareness on telecommunications – Improve public accessibility to services through the development and use of m-services (Ministry of Transport and communication - Maldives, 2006)
2014 National Broadband Policy	Policies include Providing basic broadband services Maintaining the quality of broadband services Developing broadband network infrastructure Content development and increasing the number of services provided through internet Developing broadband services to a global level (*National Broadband policy 2014-2018*, n.d.) “The broadband policy covers the period 2014-2018 and calls for broadband to be made available on all inhabited islands, for ISPs to offer an entry-level broadband plan not exceeding 4 per cent of GDP per capita, and for 100 Mbps to be made available for all commercial and industrial centres” (International Telecommunication Union, 2018)
2015 ‘Maldives Telecommunication Law' and ‘Communications Authority of Maldives' law was ratified	Components included in the Maldives Telecommunication Law: Telecommunications controls including pricing and authorisation of facilities that provide telecommunication services. Regulating quality of services provided and protecting the confidentiality and security of service seekers. Responsibility to provide access to universal services
	(Maldives Government Gazette, 2015)
